# Implementation and User Evaluation of the SANGYAN Digital Health Platform to Enhance Knowledge About COVID-19 and Other Health Conditions: Quasi-Experimental Study

**DOI:** 10.2196/67504

**Published:** 2026-05-07

**Authors:** Ashish Joshi, Ashruti Bhatt, Surapaneni Krishna Mohan, Gunjan Malhotra, Doilyn Oliveira, Saravanavel Kalpana Revathi, Ashoo Grover

**Affiliations:** 1 School of Public Health, University of Memphis, Robison Hall 3825 DeSoto Avenue, Memphis, TN, 38152, United States, 1 443 570-6018; 2 Foundation of Healthcare Technologies Society, Delhi, India; 3 Panimalar Medical College Hospital & Research Institute, Chennai, India; 4 Indian Council of Medical Research, Delhi, India

**Keywords:** podcast, usability system, client satisfaction, misinformation, COVID-19, SARS-CoV-2, pandemic, SANGYAN podcast, evidence-based, digital health intervention, usability, health literacy, REALM scale, System Usability Scale, SUS, client satisfaction questionnaire

## Abstract

**Background:**

The spread of misinformation during the COVID-19 pandemic highlighted the importance of evidence-based information. The SANGYAN podcast promotes evidence-based knowledge on health-related issues in multiple languages in a simple, cost-effective, and concise manner. This provides individuals access to the appropriate information in an accessible manner.

**Objective:**

The study’s goal is to assess user preferences for health information on a digital health platform designed to address COVID-19 misinformation.

**Methods:**

SANGYAN was developed by integrating the principles of social cognitive theory and information processing theory. The SANGYAN podcast was created to promote the importance of evidence-based information in order to address the spread of misinformation. The study design was a quasi-experimental study; prior to introducing the SANGYAN podcast, participants’ sociodemographic information was collected, and health literacy was assessed using the Rapid Estimate of Adult Literacy in Medicine, Revised scale. After listening to the podcast, participants were interviewed about its usability, and they completed the System Usability Scale and the Client Satisfaction Questionnaire - 8. Data were collected from a total of 500 participants, 250 each from the Rural Health Training Center and Panimalar Medical College Hospital & Research Institute. The participants were older than 18 years when they were included. Descriptive and bivariate analyses were performed.

**Results:**

A total of 500 participants were enrolled in the study, 50% (250/500) from rural areas and 50% (250/500) from urban areas. The majority of the participants were 45 years to 64 years old (155/500, 31%), were women (289/500, 57.8%), had poor health literacy (384/500, 76.8%), and had a high school education or less than a high school certification (241/500, 48.2%). The mean overall System Usability System score was 70.9 (SD 17.73), with those aged 18 years to 24 years having the highest mean score (81.2, SD 15.48). High user satisfaction was present, with 97.6% (487/499) obtaining the desired information from the platform.

**Conclusions:**

The study revealed that the SANGYAN podcast provides information to diverse individuals, as it is multilingual, and was found useful by the participants.

## Introduction

### Background

SARS-CoV-2, a new coronavirus, spread rapidly throughout the world. More than 200 countries and territories have been impacted by COVID-19, which started in 2019 and is still having an impact [1]. Low- and middle-income countries (LMIC) were severely impacted during the pandemic in comparison with higher-income countries [2]. Along with the spread of the virus, there was arapid circulation of misleading information on social media platforms [3].

In 1918, the influenza pandemic affected one-third of the global population and resulted in 50 million deaths worldwide. During that time, medical therapies, countermeasures, and information exchange or public health platforms were primarily accomplished via telephone, mail, or person-to-person interactions [4]. Currently, with more than 2.9 billion people using social media globally on a regular basis, information sharing has increased significantly [5,6]. Digital media was the primary means of disseminating COVID-19 information [7]. At the same time, digital media has resulted in the both knowing and unknowing spread of misleading and false information, which can influence public opinions and behaviors and have serious consequences by positively or negatively manipulating the common person’s perspective [8]. Unfortunately, this has led to people trying harmful remedies and the creation of fear and mistrust [7,9].

LMIC in particular faced unique challenges due to limited resources, poor infrastructure, unregulated social media, and a lack of crisis communication, all of which contributed to obscuring knowledge of COVID-19, panic, and confusion in these regions [10,11]. Misinformation was widespread through social media platforms such as Facebook, Twitter, and online newspapers [12]. Related literature showed that misinformation can induce anxiety, fear, and mistrust in reliable organizations through the sharing of critical and negative opinions [13]. There is evidence that misleading information is likely to spread more rapidly when finding reliable knowledge is hard and when people have low trust on the information sources accessible to them. It is important to understand that COVID-19–related information and myths are not simple enough for the general public to distinguish [14].

The World Health Organization (WHO) addressed digital health as a global strategy and defined digital health as “the field of knowledge and practice associated with the development and use of digital technologies to improve health” [15]. Although digital technologies have amplified misinformation, they can also be a source of solutions [14]. One such promising approach is the use of a targeted podcast that is contextually relevant and regionally tailored.

Digital communication technologies play a vital role in health communications and can transmit health promotion into knowledge translation in communities for public health and global health practice [16,17]. Podcasts are an effective and practical strategy for delivering and disseminating quality health-related information to the public [18]. They are a valuable resource because they provide participants with unrestricted access to necessary information in a simple, effective, and concise format [19]. Several studies have investigated the acceptability or feasibility of this mode of learning, but limited research has been conducted on the use of podcasts as a knowledge-sharing medium or platform [20]. The COVID-19 pandemic increased the number of podcast users in India, which reached up to 29.3% [21]. Podcasts enable the rapid dissemination of up-to-date information and provide listeners with a wide range of perspectives (eg, patient perspective) [22]. The development of audio or visual material for a listenership that wants to hear what they want, when they want, where they want, and how they want is at the core of podcasting [23].

In response to the increasing need for efficient health communication strategies, our study introduces SANGYAN (MultimediaAppendices 1  2) as a multilingual, interactive, decision-making, educational audio (IDEA) podcast designed to deliver and disseminate evidence-based COVID-19 and other health-related information in a format that is easy to understand. It takes into consideration the health literacy and cultural and contextual relevance of the populations living in urban and rural regions of Tamil Nadu, in the southern state of India. The study assessed the usefulness of this digital health platform.

### SANGYAN Podcast as a Digital Health Platform to Deliver Evidence-Based Health Information

**Figure 1.**
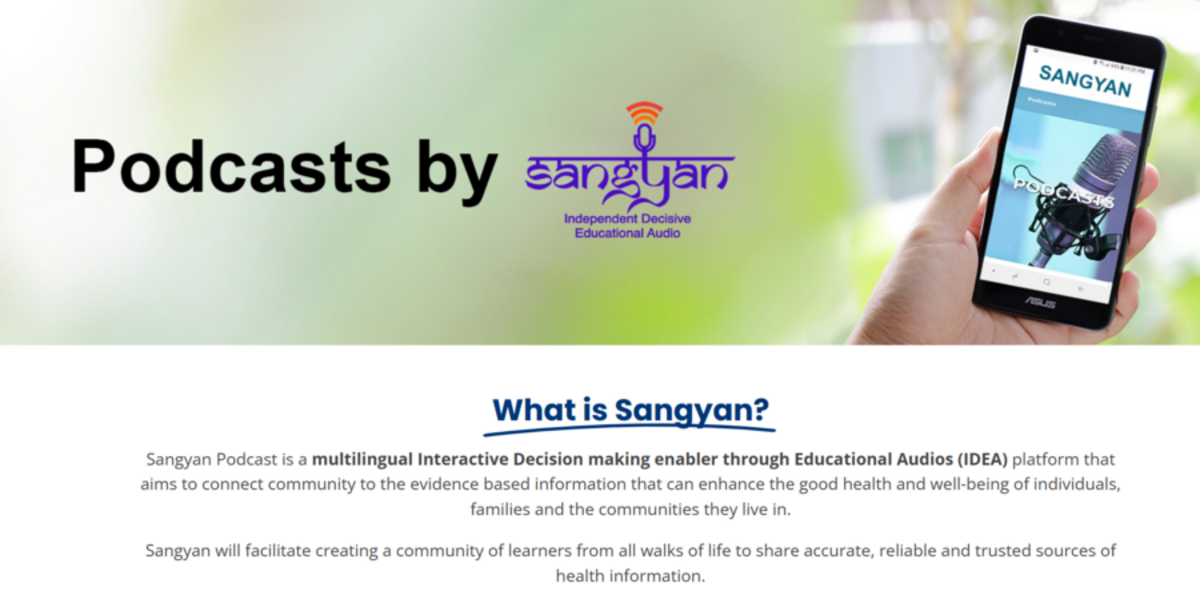
A multilingual, interactive, decision-making, educational audio (IDEA) podcast called SANGYAN[28].

The importance of leveraging digital technologies to fight against infodemics has been well recognized. Recommendations include creating educational materials, increasing the dissemination of evidence-based science, correcting behaviors, and encouraging healthy practices to address current misconceptions. SANGYAN is one such podcast-based digital platform that is evidence-based and encourages healthy practices (Figure 1) [24]. Previous studies have also recognized podcasts as a suitable source to disseminate information to improve the knowledge of their participants; similarly, SANGYAN aims to debunk misinformation at a large scale [25-27]. The SANGYAN podcast study engaged 500 study participants, 250 from rural and 250 from urban areas. The initiative aimed to promote accurate information dissemination to the public to prevent misinterpretation. The podcast does not require internet access, making it suitable for use in rural areas with limited connectivity. An open booth was established on the hospital premises, ensuring visibility to patients, relatives, and health care professionals visiting the facility. The study was self-funded and conducted in collaboration with Panimalar Medical College Hospital & Research Institute (PMCHRI).

Design and development of SANGYAN [28] involved creating a multilingual, IDEA podcast aiming to deliver evidence-based health information to individuals and the community in a clear, reliable, easy-to-understand, and culturally relevant manner. Additionally, listeners can access it easily, as it offers a free subscription and free audio download service [28] as an innovative multilingual platform designed to prevent the spread of misinformation and provide accurate information to the public. The platform was developed by a multidisciplinary team including experienced health care professionals such as nutritionists, public health professionals, and medical professionals who contributed comprehensive knowledge on various topics tailored to the needs and requirements of the community. Additionally, the communication team was responsible for collecting and disseminating health care information as well as editing audio content, while the informatics team ensured technical compliance and quality standards. Some episodes feature expert-to-expert discussions, while most include audience questions, making the SANGYAN podcasts an engaging and interactive educational tool.

SANGYAN was developed combining the principles of social cognitive theory (SCT) and information processing theory. SCT is a contemporary learning theory positing interactions among behavioral, social, and environmental factors that collectively influence human behavior. It provides a framework for understanding how individuals respond to and engage with learning opportunities, motivation, and self-regulation. In our context, we applied this theory to address societal misconceptions about various noncommunicable diseases (NCDs), COVID-19, and related health issues, with the goal of increasing public awareness. By leveraging the principles of SCT, we aimed to improve environmental factors by providing evidence-based information and enhancing knowledge, thereby counteracting misinformation [29-31]. Information processing theory aims to elucidate the mechanisms underlying human information processing, rather than solely describing behavioral responses to environmental stimuli. It posits that cognitive development is not merely a sequential progression toward a specific outcome but is also modulated by internal thoughts, prior experiences, sensory input, and perceptual processes. Additionally, it highlights the significant role of social and cultural factors in shaping cognition and learning paradigms. In addition, using the principles of information processing theory, it uses the fact that listening to podcasts helps process the information as a sensory input, which is further processed into short-term memory then saved as a long-term memory in the brain due to the fact that the information is important [32-34].

SANGYAN consists of podcasts broadly on the categories of COVID-19, NCDs, and menstrual practices. SANGYAN provides evidence-based information created by public health experts for all health topics such as diabetes, hypertension, stress, and obesity in an interactive, multilingual way. Currently, SANGYAN is available in English, Tamil, Telugu, Hindi, Gujarati, Kannada, and Malayalam.

This study represents a novel approach with the aim of addressing the critical impact of health misinformation, especially during the COVID-19 pandemic, by establishing the effectiveness of the multilingual podcast SANGYAN as a digital health tool for disseminating evidence-based information across various educational backgrounds and socioeconomic groups. Unlike traditional methods or internet-based methods, our study aimed to use podcasts to increase access to health care information without the use of the internet, even in most rural areas, in addition to being user-friendly and culturally appropriate. The quasi-experimental design addresses three key challenges: health equity, knowledge misinterpretation, and accessibility barriers. This study uniquely highlights the potential of audio-based learning systems, especially among a population with poor literacy and who is digitally naive, for enhancing equitable access to reliable health information.

## Methods

### Study Design

This pre-post, quasi-experimental study was conducted from June 2022 through August 2022 in the PMCHRI and Rural Health Training Centre (RHTC) in Chennai. Individuals present at these locations were approached and provided with a briefing of the study, and those who consented were included as participants. Each participant enrolled in the study was oriented on how to use the platform, after which the participants self-navigated the platform, selected any topic of interest, and listened to the audio podcast in a multilingual setting, based on their interest, relevance, and preferences including preferred language.

For this study, using the intervention was defined as accessing and listening to the SANGYAN podcast at least once as well as completing the postuse questionnaire. The SANGYAN platform is an open digital health platform. The podcast covered several COVID-19 topics, with all evidence-based health information created by public health experts in an interactive, multilingual format. The goal was to deliver and disseminate COVID-19–related health information to individuals and communities living in urban and rural settings in the most accurate and reliable manner and address any misinformation they might have regarding COVID-19. Satisfaction and usefulness of the SANGYAN podcast were assessed using the Client Satisfaction Questionnaire - 8 (CSQ-8) [35] and System Usability Scale (SUS) [36].

### Study Setting

The study was conducted in the RHTC, Thiruvallur District, Tamil Nadu, and PMCHRI, Chennai, Tamil Nadu.

### Sampling and Sample Size

A total sample of 500 individuals (n=250 each at the PMCHRI and RHTC) aged at least 18 years were selected using a convenience sampling method.

### Data Collection Process

The data collectors received 1 day of intensive training on the data collection process. The data collectors received a brief orientation on how to obtain informed consent and use the health literacy scale (Rapid Estimate of Adult Literacy in Medicine, Revised [REALM-R] examiner record) [37,38], SUS [36], and CSQ-8 [35]. To ensure the study’s consistency, one episode of the SANGYAN podcast series was shared with the research team. Furthermore, the team was trained in the local Indian dialect Tamil as well as English.

### Ethical Considerations

Institutional ethical clearance was provided by the PMCHRI Institutional Human Ethics Committee (CDSCO registration number ECR/1399/Inst/TN/2020 with approval number PMCH&RI/IHEC/2021/037 dated January 13, 2021). The study was conducted according to the guidelines set forth in the Declaration of Helsinki [39].

Written informed consent was obtained from each participant. For cases of illiteracy, the consent form was read to the participant, then written consent was sought. Complete confidentiality of information collected from participants was ensured. Data security was maintained through regular backups of all computers, and data files were kept password-protected. Participants were not given special incentives in cash or kind for participation in the study. As the study contributed to knowledge, there was no risk involved in participating in the study at any time.

### Variables and Measurements

Data were gathered in 2 stages. The first stage occurred prior to listening to the podcast and included data collection regarding sociodemographic characteristics, health literacy, and any prior use of podcasts or other digital medium for acquiring health information. The second stage included the collection of information after the participants listened to the podcast. This included the SUS to assess the usability and acceptance of the SANGYAN podcast and CSQ-8 to assess satisfaction with using the SANGYAN podcast as a digital health intervention to acquire evidence-based, accurate, and reliable health information in a format that is easy to understand. Individuals were asked to provide their feedback after listening to the same episode of the SANGYAN podcast to ensure consistency. Each of these variables are described in detail in the following paragraphs and in Multimedia Appendix 3.

Sociodemographic information included age, gender, education level, religion, region of residence, and employment status.

The REALM-R examiner record was also used to evaluate participants’ health literacy. It is an updated tool for estimating adult literacy in medicine. It is an 11-item word recognition test that is used to identify people who are at risk of having poor literacy skills [37,38].

To assess the use of podcasts or other media for health information, participants were asked about their prior use of podcasts to acquire health information.

The SUS measures the acceptance and usability of a system using a subjective assessment of 10 usability statements. Responses consist of 5 options, from strong agreement to strong disagreement [36]. The SUS scores were calculated with reference to the guidance outlined by Sauro [40]. The scores for each question were converted to a new number (ie, the questionnaire consisted of 10 questions that are answered on a 5-point Likert scale ranging from strongly disagree to strongly agree). On the SUS, odd-numbered statements (1, 3, 5, 7, 9) are positively expressed, and even-numbered statements (2, 4, 6, 8, 10) are negatively expressed. The responses were summed then multiplied by 2.5 to convert the original scores, which range from 0 to 40, to a range of 0 to 100. We interpreted the SUS scores using a cutoff of 68, with a score greater than 68 indicating average performance and a score less than 68 considered to be below average.

User satisfaction was assessed using the CSQ-8, which consists of 8 items and assessed general satisfaction of individuals after using the podcast [35]. The questions collect respondents’ opinions and conclusions about services they have received. Response options differ by item, but they are all based on a 4-point scale.

### Data Analysis

Descriptive analysis was conducted to outline the percentages and frequencies of the variables. Exploratory bivariate analysis was conducted to understand the correlations between sociodemographic features and their perceptions, the usefulness of the podcast, and acceptance of the podcast. No further adjusted nor multivariate analyses were conducted. A *P* value <.05 was considered significant, with 95% CIs. also considered All analyses were performed using SPSS v.24 (IBM Corp).

## Results

### Sociodemographic Details of the Study Participants

The study involved 500 participants, with 250 each from the PMCHRI, Chennai, Tamil Nadu (urban setting), and the RHTC, Thiruvallur District (rural setting). The majority of the participants (155/500, 31%) were in the age group of 45 years to 64 years old and were women (289/500, 57.8%). In addition, 11.8% (59/500) had no formal education, 44.6% (223/500) were employed, and more than one-half (384/500, 76.8%) had poor health literacy (Table 1). Rural and urban comparisons showed that a higher percentage of participants from rural settings had no formal education than those living in urban settings (45/250, 18% versus 14/250, 5.6%). A similar disparity in health literacy status was observed, with a higher proportion of participants living in rural settings having poor health literacy than those living in urban settings (217/250, 86.8% versus 167/250, 66.8%; Table 1).

**Table 1.** Sociodemographic characteristic of the participants according to region of residence.

Variables		Total sample (N=500), n (%)	Rural-dwelling participants (n=250), n (%)	Urban-dwelling participants (n=250), n (%)
Age group (years)				
18‐24		76 (15.2)	26 (10.4)	50 (20)
25‐35		138 (27.6)	76 (30.4)	62 (24.8)
36‐44		105 (21)	49 (19.6)	56 (22.4)
45‐64		155 (31)	87 (34.8)	68 (27.2)
≥65		26 (5.2)	12 (4.8)	14 (5.6)
Gender				
Male		211 (42.2)	97 (38.8)	114 (45.6)
Female		289 (57.8)	153 (61.2)	136 (54.4)
Educational level				
No formal education		59 (11.8)	45 (18)	14 (5.6)
High school or less than high school certification		241 (48.2)	148 (59.2)	93 (37.2)
Graduate and higher		168 (33.6)	43 (17.2)	125 (50)
Intermediate or diploma		32 (6.4)	14 (5.6)	18 (7.2)
Employment status				
Employed		253 (50.6)	110 (44)	143 (57.2)
Homemaker		110 (22)	78 (31.2)	32 (12.8)
Unemployed		98 (19.6)	50 (20)	48 (19.2)
Other (retired, n=14; student, n=24; lost job due to COVID-19, n=1)		39 (7.8)	12 (4.8)	27 (10.8)
Religion				
Christian		50 (10)	21 (8.4)	29 (11.6)
Hindu		434 (86.8)	223 (89.2)	211 (84.4)
Muslim		10 (2)	4 (1.6)	6 (2.4)
Unwilling to disclose		6 (1.2)	2 (0.8)	4 (1.6)
Health literacy (REALM-R^a^)				
Adequate health literacy		116 (23.2)	33 (13.2)	83 (33.2)
Poor health literacy		384 (76.8)	217 (86.8)	167 (66.8)

a REALM-R: Rapid Estimate of Adult Literacy in Medicine, Revised.

### Assessment of Health Literacy Using the REALM-R

The REALM-R was used to assess participants’ familiarity with medical jargon and to identify those at risk of poor health literacy. According to REALM-R grading, those who obtained a score ≤6 were considered to have poor health literacy, whereas those with a score >6 were considered to have adequate health literacy [38]. Table 1 shows that, overall, 76.8% (384/500) of the respondents found it challenging to read the REALM-R chart’s medical terminologies, while 23.2% (116/500) of respondents had adequate health literacy levels. The table also shows differences in health literacy levels between rural and urban settings; 86.8% (217/250) of rural residents and 66.8% (167/250) of urban residents had poor health literacy.

### Difference Between the Use of the Podcast or Other Medium to Access Relevant Health Information

Approximately 89.6% (443/500) of the total sample reported that they had never used nor heard of a podcast, with 81.2% (203/250) of urban residents and 96% (240/250) of rural residents indicating the same. Only 17.6% (44/250) of urban-dwelling participants and 3.2% (8/250) of rural-dwelling participants used podcasts to access relevant health information. Around 90.4% (24/250) of participants from rural areas and 80% (200/250) of urban-dwelling participants said they did not use any app to receive pertinent health-related information. However, 97.4% (487/500) of the study participants believed that SANGYAN podcasts gave them the necessary, pertinent information (Table 2), and 97.7% (375/379) of the participants with poor literacy felt that the podcast provided relevant information.

**Table 2.** Sociodemographic characteristics and their relationship with health information access.

Sociodemographic variables		SANGYAN podcasts provided you with relevant information (yes), n (%)	Used podcasts before (yes), n (%)	Use podcasts for health-related information (yes), n (%)	Use SMS for health-related information (yes), n (%)	Use WhatsApp for health-related information (yes), n (%)	Use any app for health-related information (yes), n (%)
Total sample (N=500)		487 (97.4)	52 (10.4)	152 (30.4)	76 (15.2)	108 (21.6)	74 (14.8)
Geographic setting							
Urban (n=250)		241 (96.4)	44 (17.6)	29 (11.6)	68 (27.2)	78 (31.2)	50 (20)
Rural (n=250)		246 (98.4)	8 (3.2)	123 (49.2)	8 (3.2)	30 (12)	24 (9.6)
Age group (years)							
18‐24 (n=76)		73 (96.1)	13 (17.1)	15 (19.7)	16 (21.1)	16 (21.1)	13 (17.1)
25‐35 (n=138)		133 (96.4)	8 (5.8)	55 (39.9)	15 (10.9)	34 (24.6)	30 (21.7)
36‐44 (n=105)		103 (98.1)	13 (12.4)	41 (39)	15 (14.3)	27 (25.7)	17 (16.2)
45‐64 (n=155)		155 (100)	17 (11)	39 (25.2)	27 (17.4)	28 (18.1)	12 (7.7)
≥65 (n=26)		23 (88.5)	1 (3.8)	2 (7.7)	3 (11.5)	3 (11.5)	2 (7.7)
Gender							
Female (n=289)		283 (97.9)	28 (69.7)	105 (36.3)	46 (15.9)	59 (20.4)	36 (12.5)
Male (n=211)		204 (96.7)	24 (11.4)	47 (22.3)	30 (14.2)	49 (23.2)	38 (18)
Educational level							
No formal education (n=59)		57 (96.6)	3 (5.1)	13 (22)	1 (1.7)	2 (3.4)	0 (0)
High school or less than high school certification (n=241)		236 (97.9)	8 (3.3)	118 (49)	29 (12)	79 (32.8)	15 (6.2)
Graduate and higher (n=168)		163 (97)	39 (23.2)	40 (23.8)	41 (24.4)	66 (39.3)	53 (31.5)
Intermediate or diploma (n=32)		31 (96.9)	2 (6.3)	4 (12.5)	5 (15.6)	7 (21.9)	6 (18.8)
REALM-R^a^							
Poor health literacy (n=384)		375 (97.7)	23 (6)	127 (33.1)	51 (13.3)	61 (15.9)	29 (7.6)
Adequate health literacy (n=116)		112 (96.6)	29 (25)	25 (21.6)	25 (21.6)	47 (40.5)	45 (38.8)
Employment status							
Employed (n=253)		246 (97.2)	34 (13.4)	67 (26.5)	45 (17.8)	69 (27.3)	55 (21.7)
Homemaker (n=110)		107 (97.3)	6 (5.5)	59 (53.6)	6 (5.5)	16 (14.5)	9 (8.2)
Unemployed (n=98)		97 (99)	7 (7.1)	24 (24.5)	16 (163)	19 (19.4)	5 (5.1)
Other (n=39: retired, n=14; student, n=24; lost job due to COVID-19, n=1)		37 (94.9)	5 (12.8)	2 (5.1)	9 (23.1)	4 (10.3)	5 (12.8)

a REALM-R: Rapid Estimate of Adult Literacy in Medicine, Revised.

### Usability of and Satisfaction With the SANGYAN Podcast

Table 3 shows the mean SUS scores with respect to sociodemographic variables. The overall mean SUS score was 70.9 (SD 17.73). By age group, participants aged from 18 years to 24 years had the highest mean score of 81.5 (SD 15.6). When compared by health literacy levels, participants with poor or adequate health literacy both demonstrated above average mean SUS scores (68.5, SD 17.2 and 78.7, SD 17.2, respectively).

**Table 3.** Mapping of sociodemographic variables with usability and satisfaction scores for the SANGYAN podcast (N=500).

Sociodemographic variables		System Usability Scale (SUS) score, mean (SD)	Score category, n (%)	
			<68 points	≥68 points
Age group (years)				
18‐24 (n=76)		81.5 (15.6)	18 (23.7)	58 (76.3)
25‐35 (n=138)		73.8 (17.6)	64 (46.4)	74 (53.6)
36‐44 (n=105)		72.1 (16)	50 (47.6)	55 (52.4)
45‐64 (n=155)		63.7 (16.5)	101 (65.2)	54 (34.8)
≥65 (n=26)		62.1 (18)	17 (65.4)	9 (34.6)
Gender				
Female (n=289)		69.3 (16.3)	151 (52.2)	138 (47.8)
Male (n=211)		73 (19.4)	99 (46.9)	112 (53.1)
Educational level				
No formal education (n=59)		60.7 (18.5)	41 (69.5)	18 (430.5)
High school or less than high school certification (n=241)		66.7 (16.6)	149 (61.8)	92 (38.2)
Graduate and higher (n=168)		80.2 (16.9)	47 (28)	121 (72)
Intermediate or diploma (n=32)		77.3 (16.1)	13 (40.6)	19 (59.4)
Health literacy				
Poor literacy (n=384)		68.5 (17.2)	216 (56.3)	168 (43.8)
Adequate literacy (n=116)		78.7 (17.2)	34 (29.3)	82 (70.7)
Region of residence				
Rural (n=250)		67.8 (17.5)	148 (59.2)	102 (40.8)
Urban (n=250)		74 (17.5)	102 (40.8)	148 (59.2)
Employment status				
Employed (n=253)		72.2 (16.2)	112 (44.3)	141 (55.7)
Homemaker (n=110)		67.1 (15.2)	67 (60.9)	43 (39.1)
Unemployed (n=98)		60.5 (17.9)	61 (62.2)	38 (38.8)
Others (n=39: retired, n=14; student, n=24; lost job due to COVID-19, n=1)		75.6 (15.8)	10 (25.6)	28 (71.8)

### Participant Satisfaction With SANGYAN: CSQ-8

Table 4 shows the percentages of participants who were satisfied with SANGYAN, where scores of 1 or 2 indicate dissatisfaction and scores of 3 or 4 indicate satisfaction. Of the participants, 94.8% (474/500) were satisfied with the quality of SANGYAN, and 97.4% (487/500) received the kind of information they desired from SANGYAN. In addition, 93% (465/500) of the participants were likely to use SANGYAN again, and 97% (485/500) of the participants would recommend SANGYAN to their friends.

**Table 4.** Client Satisfaction Questionnaire - 8 (CSQ-8) scores for satisfaction levels when using the SANGYAN podcast (N=500).

CSQ-8 questions	Score, n (%)			
	1	2	3	4
How would you rate the quality of service you received?	7 (1.4)	16 (3.2)	237 (47.4)	237 (47.4)
Did you get the kind of service you wanted?	0 (0)	12 (2.4)	211 (42.2)	276 (55.2)
To what extent has our service met your needs?	1 (0.2)	37 (7.4)	219 (43.8)	242 (48.4)
If a friend were in need of similar help, would you recommend our service to him or her?	5 (1)	9 (1.8)	174 (34.8)	311 (62.2)
How satisfied are you with the amount of help you received?	49 (9.8)	19 (3.8)	233 (46.6)	198 (39.6)
Have the services you received helped you to deal more effectively with your problems?	0 (0)	11 (2.2)	179 (35.8)	309 (61.8)
In an overall, general sense, how satisfied are you with the service you received?	5 (1)	14 (2.8)	203 (40.6)	271 (54.2)
If you were to seek help again, would you come back to our service?	9 (1.8)	18 (3.6)	138 (27.6)	327 (65.4)

### Usability of and Satisfaction With the SANGYAN Podcast as a Digital Health Platform

**Figure 2.**
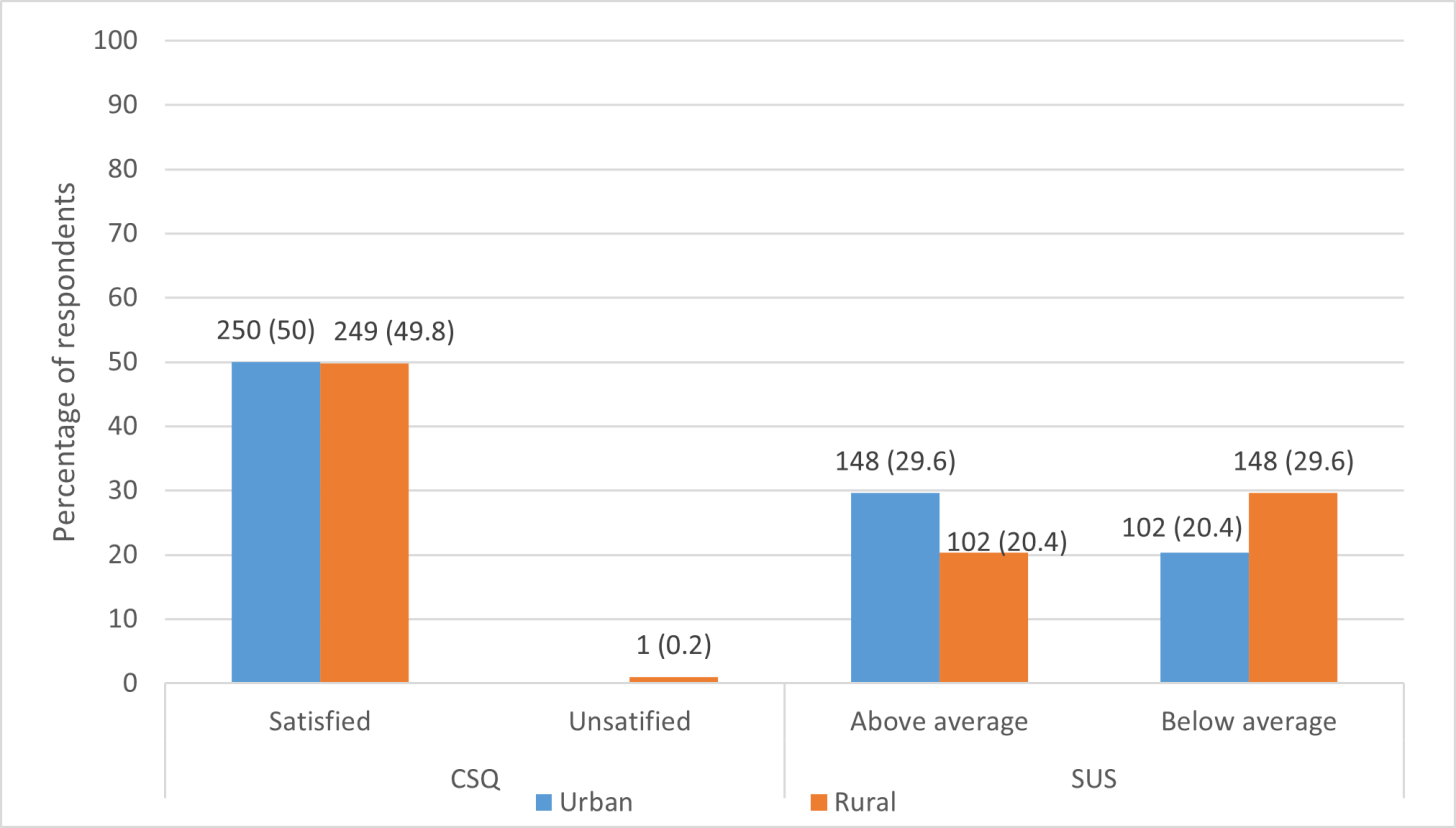
Satisfaction with and usability of the SANGYAN podcast based on results from the Client Satisfaction Questionnaire (CSQ) and System Usability Scale (SUS), respectively, compared between urban and rural residents.

Figure 2 illustrates the usability of and satisfaction with the SANGYAN podcast as a digital health platform to obtain evidence-based health information in a multilingual, interactive format that is easy to understand. Of the individuals who were asked to listen to an episode of the SANGYAN podcast, 99.8% (499/500) expressed their satisfaction with it as a platform to receive appropriate health information at the right time.

### Associations Between Sociodemographic Characteristics and SUS and CSQ-8 Scores

Table 5 shows the associations between the sociodemographic variables and SUS and CSQ-8 scores, with respect to region of residence. Above average system use was more prevalent in respondents aged 25 years to 35 years (74/250, 29.6%) than in those aged 18 years to 24 years (58/250, 23.2%), and the relationship with age groups was statistically significant (χ^2^_4_=38.7, *P*<.001). Above average ratings for system usability were more common among women (138/250, 55.2%) than men (112/250, 44.8%; χ^2^_1_=16.9, *P*<.001). In addition, statistically significant relationships between system usability ratings educational level were present (*χ*^2^_6_=64, *P*=.001), with a greater proportion (106/250, 42.4%) of those having a graduate degree rating it above average. In addition, most of the respondents in the different age groups were highly satisfied with the SANGYAN podcast. Differences in ratings by employment status were also present (χ^2^_6_=28.07, *P*<.001). There were no significant differences in SUS scores by religion (χ^2^_3_=6.69, *P*=.08).

**Table 5.** Differences in System Usability Scale (SUS) and Client Satisfaction Questionnaire - 8 (CSQ-8) scores by sociodemographic characteristics of the study respondents (N=500).

Variables		SUS						CSQ-8			
		Above average, n (%)		Below average, n (%)		χ^2^ (*df*) ^a^	*P* value	High satisfaction, n (%)		Low satisfaction, n (%)	
		Rural (n=250)	Urban (n=250)	Rural (n=250)	Urban (n=250)			Rural (n=250)	Urban (n=250)	Rural (n=250)	Urban (n=250)
Age group (years)						38.7 (4)	<.001				
18‐24 (n=76)		20 (26.3)	38 (50)	6 (7.9)	12 (15.8)			26 (34.2)	50 (65.8)	0 (0)	0 (0)
25‐35 (n=138)		37 (26.8)	37 (26.8)	39 (28.3)	25 (18.1)			76 (55.1)	62 (44.9)	0 (0)	0 (0)
36‐44 (n=105)		14 (13.3)	41 (39)	35 (33.3)	15 (14.3)			49 (46.7)	56 (53.3)	0 (0)	0 (0)
45‐64 (n=155)		27 (17.4)	27 (17.4)	60 (38.7)	41 (26.5)			87 (56.1)	68 (43.9)	0 (0)	0 (0)
≥65 (n=26)		4 (15.4)	5 (19.2)	8 (30.8)	9 (34.6)			11 (42.3)	14 (53.8)	1 (3.8)	0 (0)
Gender						16.9 (2)	<.001				
Female (n=289)		62 (21.5)	76 (26.3)	60 (20.8)	151 (52.2)			152 (52.6)	136 (47.1)	1 (0.3)	0 (0)
Male (n=211)		40 (19)	72 (34.1)	57 (27)	42 (19.9)			97 (46)	114 (54)	0 (0)	0 (0)
Educational level						64 (6)	<.001				
No formal education (n=59)		8 (13.6)	10 (16.9)	37 (62.7)	4 (6.8)			44 (74.6)	14 (23.7)	1 (1.7)	0 (0)
High school or less than high school certification (n=241)		50 (20.7)	42 (17.4)	98 (40.7)	51 (21.2)			148 (61.4)	83 (34.4)	0 (0)	0 (0)
Intermediate or diploma (n=32)		8 (25)	11 (34.4)	6 (18.8)	7 (21.9)			14 (43.8)	18 (56.3)	0 (0)	0 (0)
Graduate and higher (n=168)		36 (21.4)	85 (50.6)	7 (4.2)	40 (23.8)			43 (25.6)	125 (74.4)	0 (0)	0 (0)
Religion^b^						6.69 (3)	.08				
Christian (n=50)		14 (28)	17 (34)	7 (14)	12 (24)			21 (42)	29 (58)	0 (0)	0 (0)
Hindu (n=434)		85 (19.6)	123 (28.3)	138 (31.8)	88 (20.3)			222 (51.2)	211 (48.6)	1 (0.2)	0 (0)

a χ2 analyses were conducted using the original category groupings, but the categories were merged in the table for clarity and readability.

b Muslim participants were not reported because religion was not significantly associated with SUS scores (*P*=.08) and the number of Muslim participants in the sample was relatively small (n=10).

### Associations Between Sociodemographic Characteristics and Health Literacy (REALM-R)

Of the 155 (155/500, 31%) participants in this study who were aged from 45 years to 64 years, 87.7% (136/155) had poor health literacy (Table 6), representing the largest proportion even though the younger age groups (ie, 18‐24 years and 25‐35 years) had more limited health literacy regarding medical terminologies than the older population. There were significant associations between levels of health literacy and age groups (χ^2^_4_=25.4, *P*<.001). According to the evaluation using the REALM-R examiner record, 66.8% (167/250) of individuals from urban regions had poor health literacy, as did 86.8% (217/250) of those in rural areas. In addition, there was a significant association between health literacy (REALM-R) and regions of residence (χ^2^_1_=28.1, *P*<.001). However, the difference in the REALM-R score between genders was not statistically significant (χ^2^_1_=0.401, *P*=.53). The association between REALM-R grades and education status was statistically significant (χ^2^_6_=167, *P*<.001).

**Table 6.** Associations between the sociodemographic variables and Rapid Estimate of Adult Literacy in Medicine, Revised (REALM-R) grades.

Variables		REALM-R grade, n (%)		*χ*^2^ (*df*)^a^	*P* value
		Adequate health literacy (n=116)	Poor health literacy (n=384)		
Age group (years)				25.4 (4)	<.001
18‐24 (n=76)		27 (35.5)	49 (64.5)		
25‐35 (n=138)		45 (32.6)	93 (67.4)		
36‐44 (n=105)		21 (20)	84 (80)		
45‐64 (n=155)		19 (12.3)	136 (87.7)		
≥65 (n=26)		4 (15.4)	22 (84.6)		
Gender				0.401 (1)	.53
Female (n=289)		70 (24.2)	219 (75.8)		
Male (n=211)		46 (21.8)	165 (78.2)		
Region of residence				28.1 (1)	<.001
Rural (n=250)		33 (13.2)	217 (86.8)		
Urban (n=250)		83 (33.2)	167 (66.8)		
Education level				167 (6)	<.001
No formal education (n=59)		0 (0)	59 (100)		
High school or less than high school certification (n=241)		15 (6.2)	226 (93.8)		
Graduate and above (n=168)		95 (56.5)	73 (43.5)		
Intermediate or diploma (n=32)		6 (18.8)	26 (81.3)		
Employment status				35.3 (6)	<.001
Employed (n=253)		78 (30.8)	175 (69.2)		
Homemaker (n=110)		14 (12.7)	96 (87.3)		
Unemployed (n=99)		10 (10.2)	89 (89.9)		
Other (n=39: retired, n=14; student, n=24; unempoyed due to COVID-19, n=38)		14 (36.8)	24 (63.2)		

a χ2 analyses were conducted using the original category groupings, but the categories were merged in the table for clarity and readability.

## Discussion

### Principal Findings

In this study, 50% (250/500) of respondents were from rural areas, and 50% (250/500) were from urban areas. Interestingly, a large portion of the participants reported neither prior use (420/494, 85%) nor familiarity with podcasts (443/495, 89.5%) for gathering any kind of health-related information before this study, highlighting a considerable gap in awareness and exposure to digital learning tools across various demographics. The greatest proportion of participants was among the age group of 45 years to 64 years (155/500, 31%), followed closely by the age group of 25 years to 34 years (138/500, 27.6%). Comparatively more female participation (289/500, 57.8%) was observed, reflecting greater availability among the demographic segments. Additionally, 76.8% (384/500) of the population had a poor literacy rate, and 48.2% (241/500) of the participants had an education level of high school or less, while 11.8% (59/500) had no formal education. Our podcast effectiveness was assessed using the SUS, which revealed an overall good usability, with a mean score of 70.9. Participants aged 18 years to 24 years had the highest mean SUS score of 81.2, suggesting higher acceptance among younger users, who perceived the podcasts as more user-friendly and engaging. The CSQ evaluation showed that 94.8% of the users reported satisfaction with the quality of service received, 97.4% got the information they were seeking, 93% of the participants were likely to use SANGYAN again, and 97% would recommend SANGYAN to their friends and family, highlighting high user trust and community-oriented impact. Holistically, the SANGYAN app was well-received and perceived as a useful and credible source of health information. Similar findings were reported in recent studies that also demonstrated acceptability and effectiveness of mobile and app-based interventions for enhancing and improving health-related behaviors and user engagement among individuals with chronic conditions [41,42]. The exploratory subgroup analyses suggested that younger, female, and more educated participants found the platform more usable and satisfactory, highlighting the influence of demographic and literacy factors on digital health engagement.

In the twenty-first century, the SARS-CoV-2 pandemic affected millions of individuals globally. During this age of global communication, misleading information spreads rapidly, and this can potentially lead to unreliable sources of information overwhelming the general population [43]. A previous study in Ghana showed that podcast or voice messages are reliable sources of information against misinformation [44].

In our study, poor health literacy was observed in 76.8% (384/500) of the participants, among which 56.5% (217/384) were from rural regions and 43.5% (167/384) were from urban regions. A previous study in India also demonstrated poor health literacy, at a rate of 77% [45]. Previous studies have also reported poor health literacy in the following countries: Bulgaria (62%), Spain (58%), Suriname (95%), Saudi Arabia (54%), and South Africa (68%) [40,43-45]. In contrast, adequate health literacy was reported for the Netherlands (71%), Ireland (59%), Greece (55%), and Poland (56%). In Suriname, better health literacy was demonstrated in urban settings [46].

Further, health literacy was significantly associated with employment status, showing that unemployed people and retired people were more likely to demonstrate higher levels of poor literacy. Similarly, a previous study in Denmark found poor health literacy levels among unemployed people [47]. Health literacy also had a significant association with education levels; participants demonstrating adequate literacy were likely graduates or professionals. Similar to this, a study in Jordan showed that participants demonstrating adequate literacy were likely highly educated [48].

SANGYAN was considered a source of relevant health information by 98% of the participants, and 99.8% of the participants were satisfied with the usage of the SANGYAN podcast. Similarly, previous studies in India and the United States found that podcasts were relevant and provided the necessary information [25,26]. Studies in the United States also found that its users were satisfied with the use of podcasts [49,50].

This study showed a significant association between health literacy and SUS scores, such that a person demonstrating poor health literacy was likely to have a below average SUS score. In contrast, a previous study in the United States reported no significant association between health literacy and SUS scores for digital health–based platforms [50]. Our study showed that participants in rural settings were likely to score below average, whereas in a previous study conducted in rural settings of the United States, participants scored an average of 81 points, which indicates above average usability [51].

The participants from rural settings found the SANGYAN podcast to be a satisfactory source of health information. Similarly, a study in rural Senegal recorded satisfactory responses for voice message–based platforms [27]. The poor health literacy demonstrated among participants and their satisfactory response to the SANGYAN podcast emphasize the use of podcasts to deliver health information to participants who experience difficulty reading. Based on previous studies, podcasts are practical, easy to use, and educational [48,52].

Further, in contrast to existing literature from studies conducted in India, Uganda, and Malaysia that showed podcasts’ potential to be used as educational tools predominantly with students [53-57], this study showed that podcasts are a viable tool for information delivery across all age groups. It demonstrated that acceptance of and satisfaction with podcast use are not limited to any particular age group.

Existing studies of methods to tackle infodemics used the traditional approach of debunking myths. Although this is effective, it predominantly uses graphics. As a tool against infodemics, SANGYAN is not only evidence-based but also available in various languages. Because it is delivered in an audio format, it caters to the population with poor literacy levels [58,59].

A study reviewed various digital tools used to curb misinformation through mobile apps and official government and mass media websites [60]. Participants in another study indicated that the provision of health-related information in languages other than English would have been used [61]. With podcasting having a large audience in India, this can be one digital tool to combat misinformation. Use of podcasts for educational purposes in the global and Indian contexts has increased, making SANGYAN an ideal platform [62-67]. The results of this study demonstrated high participant satisfaction and acceptance, suggesting that SANGYAN presents an ideal multilingual approach. With the results of this study showing high satisfaction and acceptance from participants, SANGYAN could be an ideal multilingual tool to combat the rapid spread of misinformation and disinformation. Further, it helps connect communities to evidence-based information that enhances the good health and well-being of individuals, families, and the communities in which they live.

The strength of this study is that it used a digital platform as a medium to impart information, and it shared information in the form of audio rather than text, catering to a wider range of people. The study also provided podcasts through existing kiosks in rural and urban health centers, making it accessible to people without the internet or a smartphone. The information the podcast provides is evidence-based and from a reliable source, hence enabling it to tackle misinformation. Before starting the data gathering procedure, the data collectors underwent a training session. The findings of the study add to the global evidence that well-designed, user-centric, multilingual tools can improve public health engagement and enhance self-caring practices in limited-resource settings [41,42,68].

### Limitations

Our study had a few limitations. First, since we used convenience sampling, participants were recruited based on availability rather than a random selection method. Moreover, the study used a single-arm design and therefore lacked a control group to compare the findings. The research was limited to the Tamil Nadu area, limiting the external validity and generalizability of the findings to a broader population.

Last, the study did not evaluate the long-term retention of knowledge or sustained behavioral impact delivered through the podcasts. Future research studies in this domain should bridge these gaps in knowledge to strengthen the scientific evidence. This would be beneficial for observing the impact of a podcast-based digital health platform on the issue of misinformation and disinformation.

### Conclusions

This study established the acceptability of and satisfaction with the SANGYAN podcast platform among rural and urban areas. The findings emphasize the significance of creating an adaptable platform that enables people with low health literacy to connect to topics relevant to their health, by listening to clear, dependable, and brief podcast-delivered information. SANGYAN podcast could be an important source of information that can be used to more effectively educate people. Additionally, it can be used to reduce misinformation and help with the self-management of COVID-19, communicable diseases, and NCDs. This would also help to enhance the acceptability of podcasts as a source of health-related information.

## Supplementary material

10.2196/67504 Multimedia Appendix 1 Podcast image showing topics and languages.

10.2196/67504 Multimedia Appendix 2 Podcast image showing episodes and filters.

10.2196/67504 Multimedia Appendix 3 SANGYAN tool.

## Abbreviations

CSQ-8: Client Satisfaction Questionnaire - 8
IDEA: interactive, decision-making, educational audio
LMIC: low- and middle-income countries
NCD: noncommunicable disease
PMCHRI: Panimalar Medical College Hospital & Research Institute
REALM-R: Rapid Estimate of Adult Literacy in Medicine, Revised
RHTC: Rural Health Training Center
SCT: social cognitive theory
SUS: System Usability Scale
WHO: World Health Organization

## References

[1] (2022). Weekly epidemiological update on COVID-19 - 8 February 2022, edition 78. World Health Organization.

[2] Verhagen LM, de Groot R, Lawrence CA, Taljaard J, Cotton MF, Rabie H (2020). COVID-19 response in low- and middle-income countries: don’t overlook the role of mobile phone communication. Int J Infect Dis.

[3] van der Linden S, Roozenbeek J, Compton J (2020). Inoculating against fake news about COVID-19. Front Psychol.

[4] Merchant RM, Lurie N (2020). Social media and emergency preparedness in response to novel coronavirus. JAMA.

[5] Clement J Number of global social media users 2010-2021. Statista.

[6] Paules CI, Marston HD, Fauci AS (2020). Coronavirus infections-more than just the common cold. JAMA.

[7] Rastogi A, Syed S, Sharma T (2021). Enhancing the health coverage in India by empowering the corona warriors through educational intervention. Int J Community Med Public Health.

[8] R J, D B, waran K (2020). Social media reigned by information or misinformation about COVID-19: a phenomenological study. SSRN Journal.

[9] Evanega S, Lynas M, Adams J, Smolenyak K, Insights CG (2020). Coronavirus misinformation: quantifying sources and themes in the COVID-19 ‘infodemic’ (preprint). Journal of Medical Internet Research.

[10] Lau LL, Hung N, Go DJ (2020). Knowledge, attitudes and practices of COVID-19 among income-poor households in the Philippines: a cross-sectional study. J Glob Health.

[11] World Health Organization (2020). Protecting yourself and others from the spread COVID-19.

[12] Islam MS, Sarkar T, Khan SH (2020). COVID-19-related infodemic and its impact on public health: a global social media analysis. Am J Trop Med Hyg.

[13] Wang Y, McKee M, Torbica A, Stuckler D (2019). Systematic literature review on the spread of health-related misinformation on social media. Soc Sci Med.

[14] Bin Naeem S, Kamel Boulos MN (2021). COVID-19 misinformation online and health literacy: a brief overview. Int J Environ Res Public Health.

[15] Dhingra D, Dabas A (2020). Global strategy on digital health. Indian Pediatr.

[16] Dadaczynski K, Okan O, Messer M (2021). Digital health literacy and web-based information-seeking behaviors of university students in Germany during the COVID-19 pandemic: cross-sectional survey study. J Med Internet Res.

[17] Benedict L, Umakanthan B, Thane G, Wang W, Alhalbouni S (2021). Podcasting as a tool for health communication? The public health insight podcast and emergent opportunities. Global Health: Annual Review.

[18] Balls-Berry J, Sinicrope P, Valdez Soto M, Brockman T, Bock M, Patten C (2018). Linking podcasts with social media to promote community health and medical research: feasibility study. JMIR Form Res.

[19] Kamel Boulos MN, Wheeler S (2007). The emerging Web 2.0 social software: an enabling suite of sociable technologies in health and health care education. Health Info Libraries J.

[20] Ifedayo AE, Ziden AA, Ismail AB (2021). Podcast acceptance for pedagogy: the levels and significant influences. Heliyon.

[21] Eveland WP, Dunwoody S (2001). User control and structural isomorphism or disorientation and cognitive load?. Communic Res.

[22] Newman J, Liew A, Bowles J, Soady K, Inglis S (2021). Podcasts for the delivery of medical education and remote learning. J Med Internet Res.

[23] Jham BC, Duraes GV, Strassler HE, Sensi LG (2008). Joining the podcast revolution. J Dent Educ.

[24] Mheidly N, Fares J (2020). Leveraging media and health communication strategies to overcome the COVID-19 infodemic. J Public Health Policy.

[25] Militello L, Sezgin E, Huang Y, Lin S (2021). Delivering perinatal health information via a voice interactive app (SMILE): mixed methods feasibility study. JMIR Form Res.

[26] Murthy N, Chandrasekharan S, Prakash MP (2020). Effects of an mHealth voice message service (mMitra) on maternal health knowledge and practices of low-income women in India: findings from a pseudo-randomized controlled trial. BMC Public Health.

[27] Downs SM, Sackey J, Kalaj J, Smith S, Fanzo J (2019). An mHealth voice messaging intervention to improve infant and young child feeding practices in Senegal. Matern Child Nutr.

[28] Sangyan. Foundation of Healthcare Technologies Society.

[29] Connor M, Norman P (2005). Predicting Health Behaviour.

[30] ScienceDirect Social cognitive theory. ScienceDirect.

[31] Ab Rahim NR, Darwis N, Noh N, Asfar J, Ismail HB (2025). Exploring learning strategies through the social cognitive theory. International Journal of Research and Innovation in Social Science.

[32] Khan A, Khan S, Zia-Ul-Islam S, Khan M (2017). Communication skills of a teacher and its role in the development of the students’ academic success. Journal of Education and Practice.

[33] Bouchrika I (2025). What is information processing theory? Stages, models & limitations for 2026. Research.com.

[34] Swanson HL (1987). Information processing theory and learning disabilities: an overview. J Learn Disabil.

[35] Attkisson CC, Greenfield TK, Maruish ME (1994). The Use of Psychological Testing for Treatment Planning and Outcome Assessment.

[36] Lewis JR (2018). The System Usability Scale: past, present, and future. International Journal of Human–Computer Interaction.

[37] Dumenci L, Matsuyama RK, Kuhn L, Perera RA, Siminoff LA (2013). On the validity of the Rapid Estimate of Adult Literacy in Medicine (REALM) scale as a measure of health literacy. Commun Methods Meas.

[38] Assessment tools. Adult Meducation.

[39] World Medical Association (2013). World Medical Association Declaration of Helsinki: ethical principles for medical research involving human subjects. JAMA.

[40] Sauro J (2010). A Practical Guide to Measuring Usability.

[41] Chen D, Zhang H, Wu J (2023). Effects of an individualized mHealth-based intervention on health behavior change and cardiovascular risk among people with metabolic syndrome based on the behavior change wheel: quasi-experimental study. J Med Internet Res.

[42] Kerr D, Ahn D, Waki K, Wang J, Breznen B, Klonoff DC (2024). Digital interventions for self-management of type 2 diabetes mellitus: systematic literature review and meta-analysis. J Med Internet Res.

[43] Nelson T, Kagan N, Critchlow C, Hillard A, Hsu A (2020). The danger of misinformation in the COVID-19 crisis. Mo Med.

[44] Willcox M, Moorthy A, Mohan D (2019). Mobile technology for community health in Ghana: is maternal messaging and provider use of technology cost-effective in improving maternal and child health outcomes at scale?. J Med Internet Res.

[45] U P R, Belman M, Kamath A, B U, Shenoy K A, A L U (2013). Evaluation of health literacy status among patients in a tertiary care hospital in coastal karnataka, India. J Clin Diagn Res.

[46] Diemer FS, Haan YC, Nannan Panday RV, van Montfrans GA, Oehlers GP, Brewster LM (2017). Health literacy in Suriname. Soc Work Health Care.

[47] Abdel-Latif MMM, Saad SY (2019). Health literacy among Saudi population: a cross-sectional study. Health Promot Int.

[48] van Rensburg ZJ (2020). Levels of health literacy and English comprehension in patients presenting to South African primary healthcare facilities. Afr J Prim Health Care Fam Med.

[49] Sørensen K, Pelikan JM, Röthlin F (2015). Health literacy in Europe: comparative results of the European Health Literacy Survey (HLS-EU). Eur J Public Health.

[50] Svendsen IW, Damgaard MB, Bak CK (2021). Employment status and health literacy in Denmark: a population-based study. Int J Public Health.

[51] Nobis S, Lehr D, Ebert DD (2015). Efficacy of a web-based intervention with mobile phone support in treating depressive symptoms in adults with type 1 and type 2 diabetes: a randomized controlled trial. Diabetes Care.

[52] Martinez W, Hackstadt AJ, Hickson GB (2021). The My Diabetes Care patient portal intervention: usability and pre-post assessment. Appl Clin Inform.

[53] Wolpaw J, Toy S (2018). Creation and evaluation of an anesthesiology and critical care podcast. J Educ Perioper Med.

[54] Thirumalai M, Brown N, Niranjan S (2022). An interactive voice response system to increase physical activity and prevent cancer in the rural Alabama black belt: design and usability study. JMIR Hum Factors.

[55] Fitzpatrick AL, van Pelt M, Heang H (2019). Using targeted mHealth messages to address hypertension and diabetes self-management in Cambodia: protocol for a clustered randomized controlled trial. JMIR Res Protoc.

[56] Kaahwa M, Zhu C, Muhumuza M (2018). Investigating ICT skills and the use of audio media in distance education among teachers and students: the case of Mountains of the Moon University in Uganda. AF.

[57] Kaahwa M, Zhu C, Muhumuza M, Karemera C (2021). Differences between audio media and conventional methods regarding students’ academic performance and the influence of audio media satisfaction on their academic scores at a Ugandan university. IJIL.

[58] Mugahed W, Othman MS, Al-Rahmi WM (2013). Evaluating student’s satisfaction of using social media through collaborative learning in higher education. International Journal of Advances in Engineering & Technology.

[59] Dajani FK, Scheg AG (2015). Critical Examinations of Distance Education Transformation across Disciplines.

[60] Khoramian Tusi S, Sheikh Fathollahi M, Rahnamaye Tamrooyee F, Akbari Javar M (2015). Study of the effect of podcasting on learning and satisfaction in dental students. Journal of Mashhad Dental School.

[61] van der Linden S (2022). Misinformation: susceptibility, spread, and interventions to immunize the public. Nat Med.

[62] Challenger A, Sumner P, Bott L (2022). COVID-19 myth-busting: an experimental study. BMC Public Health.

[63] Alonso-Galbán P, Alemañy-Castilla C (2020). Curbing misinformation and disinformation in the COVID-19 era: a view from Cuba. MEDICC Rev.

[64] Lockyer B, Islam S, Rahman A (2021). Understanding COVID-19 misinformation and vaccine hesitancy in context: findings from a qualitative study involving citizens in Bradford, UK. Health Expect.

[65] Cho D, Cosimini M, Espinoza J (2017). Podcasting in medical education: a review of the literature. Korean J Med Educ.

[66] Lee C, Zhou MS, Wang ER, Huber M, Lockwood KK, Parga J (2022). Health care professional and caregiver attitudes toward and usage of medical podcasting: questionnaire study. JMIR Pediatr Parent.

[67] MacKenzie LE (2019). Science podcasts: analysis of global production and output from 2004 to 2018. R Soc Open Sci.

[68] Lee K, Chung Y, Kim JS (2024). Research trends on metabolic syndrome in digital health care using topic modeling: systematic search of abstracts. J Med Internet Res.

