# Supplementary figures and images for: Implementation and User Evaluation of the SANGYAN Digital Health Platform to Enhance Knowledge About COVID-19 and Other Health Conditions: Quasi-Experimental Study
Source: JMIR Infodemiology. 2026 May 7;6:e67504. doi: 10.2196/67504 (PMC13152227; doi:10.2196/67504)

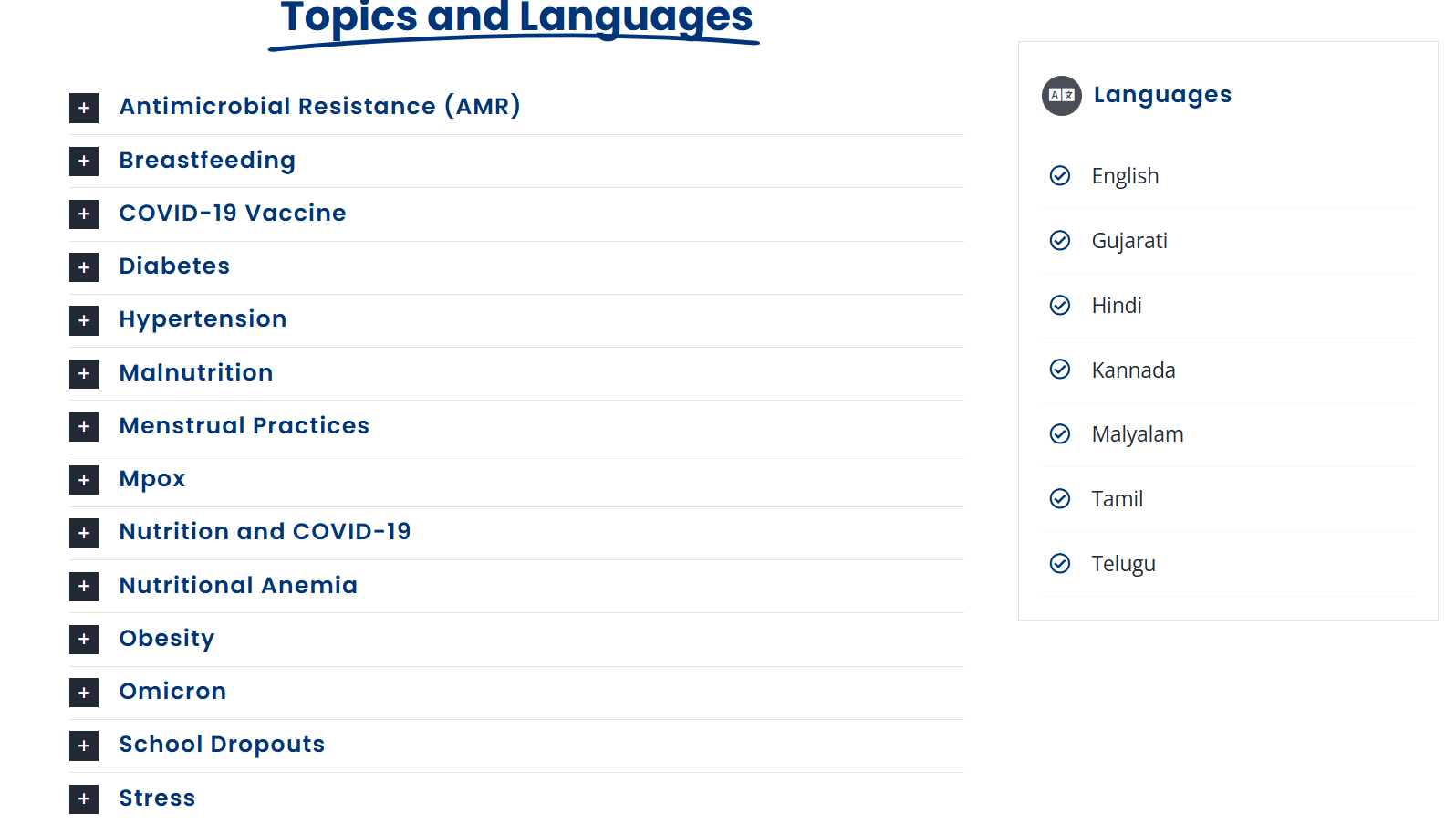

Supplement: Multimedia Appendix 1 [file infodemiology-v6-e67504-s001.png]

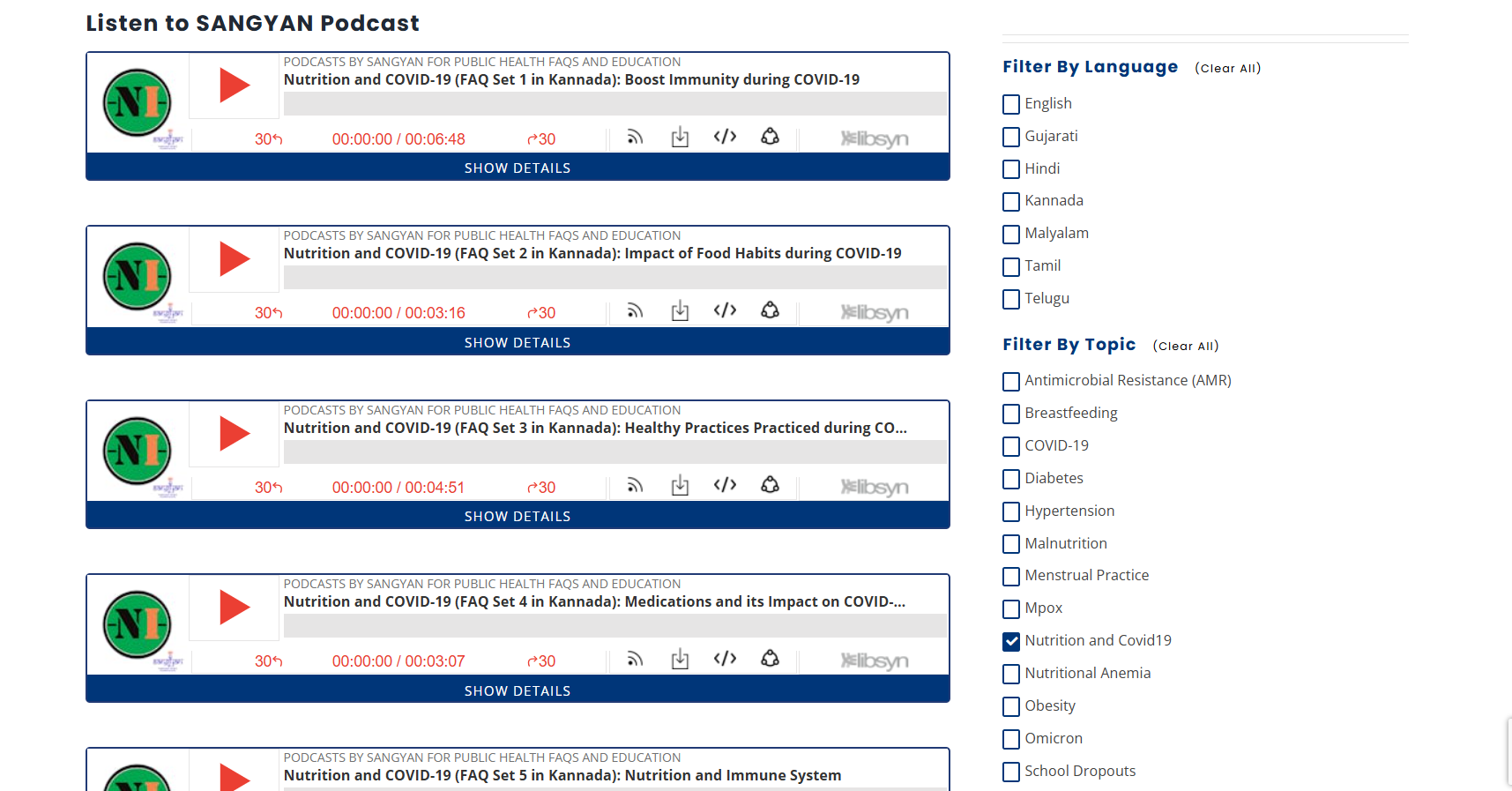

Supplement: Multimedia Appendix 2 [file infodemiology-v6-e67504-s002.png]
