# Supplementary material for: Implementation and User Evaluation of the SANGYAN Digital Health Platform to Enhance Knowledge About COVID-19 and Other Health Conditions: Quasi-Experimental Study
Source: JMIR Infodemiology. 2026 May 7;6:e67504. doi: 10.2196/67504 (PMC13152227; doi:10.2196/67504)
Supplement: Multimedia Appendix 3 [file infodemiology-v6-e67504-s003.docx]

## Multimedia Appendix 1

# Full text of the survey questionnaire.

# SURVEY CATEGORIES:

| **Annexure Number** | **Annexure Title** |
| --- | --- |
| 1. | Informed consent form |
| 2. | Socio-demographic profile |
| 3. | REALM for Health Literacy |
| 4 | System Usability Scale |
| 5 | Client Satisfaction Questionnaire (CSQ-8) |

1. **Consent form**

**Title**: To find usefulness and effectiveness of digital health intervention platform Sangyan among individuals coming to the hospital and community settings.

**PI**: Dr. Ashish Joshi

**Objective**: To investigate the levels of knowledge towards COVID-19 and other health conditions among the individuals coming to hospital and community settings and to explore the impact of Sangyan podcast as an intervention to enhance Knowledge of the individuals.

**Informed Consent**

I understand that I am being asked to participate in this study to facilitate the researchers to examine impact of podcast on knowledge of COVID-19 and other health conditions among individuals coming to hospital and community settings.

I understand that my participation in the study is voluntary and that it requires me to provide information on my socio demographics, health literacy and knowledge of COVID-19 and other health conditions. I also authorize the distribution of my picture for educational and research purpose only.

I understand that the information provided by me will be used for research purposes and the results of the study will be included in journal publications and may be presented in the conferences but will not include any identifiers and personnel information will be kept confidential

Signature of Field Staff: _____________________

Name of the Respondent with signature: ____________________

Thumb Impression __________________

Mobile/Phone: _____________________

Address:_______________________________________________________________________________________________________________________________

Date and Time: ________________________

**2. Sociodemographic profile**

| **S. No** | **Questions** |  |
| --- | --- | --- |
| **2** | **DATA COLLECTOR INFORMATION** |  |
| 2a | Date of visit | dd-mm-yyyy |
| 2b | Time of visit | _ _:_ _ hours |
| 2c | Names of Data Collection team | 1.  2.  3. |
|  | **GEOGRAPHICAL INFORMATION** |  |
| 2d | Hospital name |  |
| 2e | Pin code |  |
| 2f | Location and coordinates |  |
|  | **INDIVIDUAL PROFILE** |  |
| 2g | Name of respondent |  |
| 2h | Respondent ID |  |
|  | **SOCIO-DEMOGRAPHIC** |  |
| 2i | Age group (in years) | 1. 18-24 2. 25-35 3. 36-44 4. 45-64 5. 65+ |
| 2j | Gender | 1. Male 2. Female 3. Others, please specify.………… 4. Not willing to tell |
| 2k | Education level | 1. Illiterate 2. Primary school certification 3. Middle School certification 4. High school certification 5. Intermediate or diploma 6. Graduate 7. Profession or Honours |
| 2l | Religion | 1. Hindu 2. Sikh 3. Christian 4. Muslim 5. Other, please specify___________ 6. Not willing to tell |
| 2m | Region of Residence | 1. Urban 2. Rural 3. Slum |
| 2n | Employment Status | 1. Student 2. Unemployed 3. Employed 4. Self-employed 5. Retired 6. Homemaker 7. Lost job due to COVID-19 |

**3. REALM (Health literacy)**

fat fatigue ____

flu directed ____

pill colitis ____

allergic ____ constipation ____

jaundice ____ osteoporosis ____

anemia ____

Fat, Flu, and Pill are not scored.

Place a check mark next to each word the person pronounces correctly, and an "X" next to each work the person does not attempt or mispronounces.

4. **System Usability Scale**

| SYSTEM USABILITY SCALE | | Strongly disagree | Disagree | Neutral | Agree | Strongly agree |
| --- | --- | --- | --- | --- | --- | --- |
| 1 | I think I would like to use this program frequently |  |  |  |  |  |
| 2 | I found the program unnecessarily complex |  |  |  |  |  |
| 3 | I thought the program was easy to use |  |  |  |  |  |
| 4 | I think I would need the support of a technical person to be able to use this program |  |  |  |  |  |
| 5 | I found the various programs in this program were well integrated |  |  |  |  |  |
| 6 | I thought there was too much inconsistency in this program |  |  |  |  |  |
| 7 | I would imagine that most people that most people would learn to use this program quickly |  |  |  |  |  |
| 8 | I found the program very cumbersome/awkward to use |  |  |  |  |  |
| 9 | I felt confident using the program |  |  |  |  |  |
| 10 | I needed to learn a lot of things before I could going with this system |  |  |  |  |  |

**5. Client Satisfaction Questionnaire (CSQ-8)**

1. How would you rate the quality of service you received?

 Excellent (4)

 Good (3)

 Fair (2)

 Poor (1)

2. Did you get the kind of service you wanted?

 No, definitely not (1)

 No, not really (2)

 Yes, generally (3)

 Yes, definitely (4)

3. To what extent has our service met your needs?

 Almost all of my needs have been met (4)

 Most of my needs have been met (3)

 Only a few of my needs have been met (2)

 None of my needs have been met (1)

4. If a friend were in need of similar help, would you recommend our service to him or her?

 No, definitely not (1)

 No, I don’t think so (2)

 Yes, I think so (3)

 Yes, definitely (4)

5. How satisfied are you with the amount of help you received?

 Quite dissatisfied (1)

 Indifferent or mildly dissatisfied (2)

 Mostly satisfied (3)

 Very satisfied (4)

6. Have the services you received helped you to deal more effectively with your problems?

 Yes, they helped a great deal (4)

 Yes, they helped somewhat (3)

 No, they really didn’t help (2)

 No, they seemed to make things worse (1)
7. In an overall, general sense, how satisfied are you with the service you received?

 Very satisfied (4)

 Mostly satisfied (3)

 Indifferent of mildly dissatisfied (2)

 Quite dissatisfied (1)

8. If you were to seek help again, would you come back to our service?

 No, definitely not (1)

 No, I don’t think so (2)

 Yes, I think so (3)

 Yes, definitely (4)
